# Application of Big Data Technology for COVID-19 Prevention and Control in China: Lessons and Recommendations

**DOI:** 10.2196/21980

**Published:** 2020-10-09

**Authors:** Jun Wu, Jian Wang, Stephen Nicholas, Elizabeth Maitland, Qiuyan Fan

**Affiliations:** 1 Dong Fureng Institute of Economic and Social Development Wuhan University Wuhan China; 2 Dong Fureng Institute of Economic and Social Development Wuhan University Beijing China; 3 Australian National Institute of Management and Commerce Sydney Australia; 4 Newcastle Business School University of Newcastle Newcastle Australia; 5 School of Management University of Liverpool Liverpool United Kingdom

**Keywords:** big data, COVID-19, disease prevention and control

## Abstract

**Background:**

In the prevention and control of infectious diseases, previous research on the application of big data technology has mainly focused on the early warning and early monitoring of infectious diseases. Although the application of big data technology for COVID-19 warning and monitoring remain important tasks, prevention of the disease’s rapid spread and reduction of its impact on society are currently the most pressing challenges for the application of big data technology during the COVID-19 pandemic. After the outbreak of COVID-19 in Wuhan, the Chinese government and nongovernmental organizations actively used big data technology to prevent, contain, and control the spread of COVID-19.

**Objective:**

The aim of this study is to discuss the application of big data technology to prevent, contain, and control COVID-19 in China; draw lessons; and make recommendations.

**Methods:**

We discuss the data collection methods and key data information that existed in China before the outbreak of COVID-19 and how these data contributed to the prevention and control of COVID-19. Next, we discuss China’s new data collection methods and new information assembled after the outbreak of COVID-19. Based on the data and information collected in China, we analyzed the application of big data technology from the perspectives of data sources, data application logic, data application level, and application results. In addition, we analyzed the issues, challenges, and responses encountered by China in the application of big data technology from four perspectives: data access, data use, data sharing, and data protection. Suggestions for improvements are made for data collection, data circulation, data innovation, and data security to help understand China’s response to the epidemic and to provide lessons for other countries’ prevention and control of COVID-19.

**Results:**

In the process of the prevention and control of COVID-19 in China, big data technology has played an important role in personal tracking, surveillance and early warning, tracking of the virus’s sources, drug screening, medical treatment, resource allocation, and production recovery. The data used included location and travel data, medical and health data, news media data, government data, online consumption data, data collected by intelligent equipment, and epidemic prevention data. We identified a number of big data problems including low efficiency of data collection, difficulty in guaranteeing data quality, low efficiency of data use, lack of timely data sharing, and data privacy protection issues. To address these problems, we suggest unified data collection standards, innovative use of data, accelerated exchange and circulation of data, and a detailed and rigorous data protection system.

**Conclusions:**

China has used big data technology to prevent and control COVID-19 in a timely manner. To prevent and control infectious diseases, countries must collect, clean, and integrate data from a wide range of sources; use big data technology to analyze a wide range of big data; create platforms for data analyses and sharing; and address privacy issues in the collection and use of big data.

## Introduction

Big data are complex data sets that traditional data processing systems cannot efficiently and economically store, manage, or process. Compared to traditional data, big data has five “V” characteristics: volume, variety, velocity, veracity, and value [1]. In the digital economy era, data are a fundamental strategic resource for countries, enhancing the government’s social governance capacity and public service levels. Big data technology supports a wide range of health care functions, including clinical decision support, population health management, and disease monitoring [2,3]. By discovering correlations in data and understanding patterns and trends, big data technology can improve health care, save lives, and reduce health system costs. Through the analysis of patient characteristics and patient nursing costs, the most clinical cost-effective treatment methods can be determined; the application of big data analysis technology to patient files can identify individuals who may benefit from preventive care or lifestyle changes; the collection and analysis of medical procedure data can determine the most valuable patient nursing programs; and through analysis and drug treatment data, the health status of the population can be monitored and the health status of patients maximized through drug treatments [4]. In the prevention and control of pandemics, large data technology can be used for epidemic prediction, pandemic alerts, tracking and tracing of infected individuals, identifying potential pharmacological treatments, and optimal resource allocations within the health system [5-10].

Big data analytics is fast becoming a crucial component for the modeling of virus transmission, aiding infection control measures and emergency response analyses required during local or international disease outbreaks [11]. In the prevention and control of infectious diseases, previous research on the application of big data technology has mainly focused on the early warning and monitoring of infectious diseases. Four data streams are used for early warning and monitoring infectious diseases: medical health data, participatory syndromic data, internet data, and nonhealth digital data [12]. Medical health data include electronic records of medical institutions, medical insurance claims, discharge records, and death certificates. Such data can provide information about disease conditions and can be monitored at various levels or aggregated by geographic location before reporting [13-15]. Participatory syndromic data are data from crowdsources, where volunteers report a series of symptoms on their own. These data streams do not provide the confirmed infection status of specific pathogens but provide personal-level health data in almost real time [16-18]. Internet data does not depend on a specific patient or medical condition but originates from the use of internet search engines, social media, or online consumption. In addition to self-reporting health results, these data streams also provide information about health-related behaviors, including contact and travel patterns, and vaccine status, which are key elements to understanding the spread of diseases [19-24]. Nonhealth digital data mainly include social and natural factors such as weather, temperature, humidity, population movement, transportation, infrastructure, and medical environment [25-28]. With the development of computer and space technology, geographic information system remote sensing is increasingly used in infectious disease monitoring and early disease warning research because of its powerful geospatial data acquisition, management, processing, analysis, and display capabilities [29,30].

Before the outbreak of COVID-19, China mainly used infectious disease case data reports for disease early warning and monitoring. In recent years, China has been working to advance the informationization process of medical institutions, store information related to medical services in computer network systems, and accumulate a large amount of medical service data. For example, the hospital information system (HIS) is an important source of medical health data. HIS mainly includes a hospital management information system, laboratory information system (LIS), medical image archive and communication system (picture archiving and communication system), radiation information management system (radiology information system), and clinical decision support system. The electronic medical record system (EMRS) in the medical and health departments includes data on patient name, treatment data, illnesses, test results, orders, operation records, and nursing records.

After experiencing the severe acute respiratory syndrome (SARS) outbreak in 2003, the Chinese government formulated plans to identify the early signs of infectious diseases, setting in law the requirement under the Emergency Regulations for Public Health Emergencies that units that discover infectious diseases must report the disease one level up within a specified time frame. These reports contain information on patient’s name, ID number, age, occupation, residential address, date of disease onset, date of diagnosis, type of infectious disease, and route of transmission. In addition, China has established a service network that links disease control institutions, hospitals, and primary medical and health institutions. Under the National Infectious Disease Report Information Management System (NIDRIMS), institutions immediately report new infectious disease cases online. Those institutions without direct network reporting capabilities were instructed to immediately report the case to the local district-level disease control institution and complete an infectious disease report card within 2 hours. Based on the newly entered data on the direct network reporting system and the historically accumulated infectious disease data, an automatic national infectious disease early warning and surveillance information system was created in April 2008, and an infectious disease monitoring data system trailed in December 2009. For example, a doctor diagnosing a patient with tuberculosis can use the test results to extract the information needed to complete the infectious disease report from the patient’s electronic medical records, which is submitted to the NIDRIMS. NIDRIMS can query the detailed information of the case to monitor and provide an early warning of infectious diseases. The national infectious disease early warning system and surveillance system significantly facilitated the prevention and control of infectious diseases in China. In the H7N9 epidemic in 2013, although the virus spread widely and affected more than ten Chinese provinces and cities, the actual number of infected people was only 132, with 29 deaths and no medical staff infected.

After the outbreak of COVID-19, the Chinese government and social organizations actively used big data technology to prevent and control the disease. At the Scheduling Meeting on Big Data Supporting the Prevention and Control of Corona Virus Disease 2019 held by the Ministry of Industry and Information Technology on January 26, 2020, a joint antiepidemic prevention and control mechanism was proposed, and big data analysis was mobilized to guide research and predict the epidemic’s developments, including the deployment of antiepidemic work and monitoring the health of mobile personnel. On February 14, 2020, the government further ordered the use of digital technologies including big data, artificial intelligence (AI), and cloud computing to include epidemic monitoring and analysis, tracing of virus sources, epidemic prevention and treatment, and resource allocation.

Existing papers on COVID-19 big data provide limited discussion and analysis on specific big data applications [10,31]; address the application of big data in a subdivision of COVID-19 prevention and control, and discuss the application of specific types of data in the prevention and control of COVID-19 [6,7,30,32]; or mainly discuss the application methods and achievements of big data technology outside of China [8,9]. Adding to the existing literature, this paper reviews the data collection methods and data information that existed in China before the outbreak of COVID-19. We introduce China’s post–COVID-19 data collection methods and data information analyses. Based on the increased data collected, China’s experiences in applying big data technology for personnel tracking, epidemic surveillance, early warning, tracing of virus sources, drug screening, medical treatment, resource allocation, and production recovery are detailed. We also analyzed the issues, challenges, and responses encountered by China in the application of big data technology to prevent and control COVID-19 from four perspectives: data access, data use, data sharing, and data protection. Suggestions for further improvements are made from four perspectives, data collection, data circulation, data innovation, and data security, to help understand China’s response to the epidemic and to provide lessons for other countries facing the COVID-19 pandemic.

## Methods

By June 2019, the number of mobile internet users in China was 847 million [33]. In recent years, the degree of informatization by the Chinese government and various social organizations has continuously improved. China’s data collection methods and data reserves also increased. Textbox 1 summarizes the pre–COVID-19 key data collection methods and data information that helped prevent and control COVID-19, while Textbox 2 provides an outline of the newly added data collection methods and data information implemented after the outbreak of COVID-19.

**Pre–COVID-19 disease prevention and control data from government, commercial and public welfare sources and websites, academic papers, public reports, institutional reports, and fieldwork.**

**Location information data**

- Telecommunication operators
  Mobile phone signaling generates location data allowing the user’s location to be identified and tracked based on the location of the base station. The acquisition of these data requires the authorization of the Public Security Department. In addition to operators, some government departments, large internet companies, and map service providers can apply for the acquisition of these data.
  When applying for a mobile phone number, you need to provide the user’s name, ID number, residential address, and other information, which can be accessed by the operator and government departments.
- Mobile payment
  The electronic wallet is bound to the user’s ID card and mobile phone number. The payee is usually a store or an enterprise and has location information on the map. Mobile payment is widely used in China.
- Takeaway
  Can provide the user's residential address
- Online shopping
  Requires mobile phone number and residential address
- Courier
  Courier requires customer’s ID card number, mobile phone number, and residential address.
- Internet Protocol address
  Users will leave location information when they go online.

**Travel data**

- Ticket purchase information
  Purchase of train tickets, plane tickets, ferry tickets, and bus tickets require the user’s name, ID number, and mobile phone number information.
- Toll station vehicle information
  At the entrance and exit of the road, the license plate number is bound to the ID card information.
- Taxi software
  Online car-hailing software (Didi, Shenzhou special car, Shouqi car-hailing, etc) collect the departure and destination addresses.
- Map data
  Automatically locates the user’s location when running map software
- Card swipe information data
  The bus and subway provide payment records.

**Medical health data**

- Electronic medical records
  Includes data on patient name, treatment data, illnesses, test results, orders, operation records, and nursing records
- National Health Commission
  Name, ID number, age, occupation, residential address, date of onset, date of diagnosis, and type of infectious disease when encountering infectious disease cases

**Internet Data**

- Consumer data
  Online shopping
- News data
  Online news media information
- Social data
  Communicated by the public through social software
- Retrieve data
  User search history

**Government data**

- Tax data
  Tax department continues to collect
- Electricity data
  The energy department will continue to collect and record
- Legal data
  The legal department will continue to collect and record

**Newly added data collected in China after the outbreak of COVID-19 from government, commercial and public welfare sources and websites, academic papers, public reports, institutional reports, and fieldwork.**

**Location information data**

- Questionnaire data from schools and various organizations, including current residential address, through an online questionnaire (WeChat or Alipay applet)

**Consumption data**

- When purchasing special medicines (treatment of colds, fever, and coughing, and other medicines), ID number and residential address information

**Reservation data**

- To go to hospitals, parks, and other places, you need to use your ID number and mobile phone number to make an appointment

**Registration data**

- Temporary access to shopping malls, restaurants, and other public places requires registration of ID card number and mobile phone number

**Travel data**

- Questionnaire
  When you travel, you need to provide departure address and destination information.

**Patient data**

- National Health Commission
  Information on confirmed and suspected COVID-19 cases (name, ID card number, age, occupation, residence address, date of onset, date of diagnosis) by the National Health Commission

**Health status data**

- Questionnaire survey
  Daily report of health status (temperature and the presence of cold, fatigue, coughing symptoms) on time every day by internet applet
- Drug purchase information
  When purchasing special drugs (treatment of colds, fever, and coughing, and other drugs) data on name, ID, and address, and data on body temperature and cold, fatigue, and coughing symptoms
- Smart devices
  Thermal imaging human body temperature measurement devices in public places such as train stations, airports, shopping malls, hospitals, and pharmacies automatically collect data.

**Treatment data**

- Hospital
  Statistics on the treatment effect on patients with COVID-19 treated with different drugs

**Prevention and control materials data**

- The government department
  Supply and demand information, price, and quantity of various epidemic prevention materials into a system platform

**Data of designated**

- Hospitals, government, and map suppliers
  Government’s listed designated hospitals have special identification label on map

**Figure 1:**
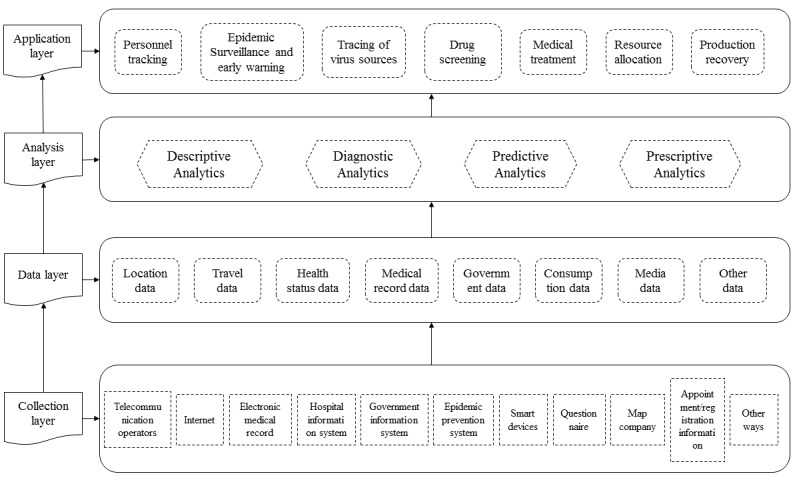
Conceptual structure chart of prevention and control of COVID-19 with big data technology.

Figure 1 sets out a diagrammatic representation of the conceptual basis of big data technology for COVID-19 prevention and control on four levels: application, analysis, data, and collection. In the next section, we discuss the key elements within each collection, data, analysis, and application layer of Figure 1.

## Results

### Location Data and Travel Data

Turning to the collection, data, analysis, and application layers in Figure 1, the scale of population migration is crucial for disease space propagation predictions, risk area identification, and control measure decisions to control infectious diseases [34]. Studies have shown that there is a close relationship between the number of train trips and the number of COVID-19 cases [35]. Before Wuhan was quarantined on January 23, 2020, more than 5 million people left Wuhan for other parts of China [36]. Internet companies used ticket purchase information and toll booth information to create population migration maps. The maps show the movement of population between cities in specific time ranges. For January 22, 2020, the day before the closure of Wuhan, the data found that 74.77% of the total number of people who moved out of Wuhan migrated to other parts of Hubei Province including 14.56% in Xiaogan City, 14.08% in Huanggang City, and 7.65% in Jingzhou City; 5.67% moved to Henan Province; and 3.24% moved to Hunan Province [37]. In the early stage of the epidemic, the travel data identified cities and regions susceptible to potential future outbreaks, allowing government organizations to make timely preparations for epidemic prevention and control.

According to the patient information data provided by the National Health Commission, internet companies and government official websites produced epidemic maps showing the number of patients diagnosed with COVID-19, the number of suspected patients, and the number of cured patients in each province and city [38]. The death toll allowed the public to keep abreast of the epidemic’s development. For example, based on a unified geographic framework, research institutions quickly absorbed and integrated geographic big data including internationally published World Health Organization data, daily family health and disease control data, professional population health platform data, Tencent site selection request data, Baidu migration data, patient spatial-temporal trajectory data, international airline data, census data, remote sensing images, and other multisource data. Multi-scale comprehensive spatial-temporal dynamic visualization technology allowed provinces, cities, counties, communities, and individual epidemic data to be unified in a spatial-temporal data visualized on a multidimensional “one map.” The map service provider identified the residential areas with confirmed or suspected cases on the map, and the map users can interrogate the information around their specific locations on the map. Individuals can avoid activities in high-risk areas, and hospitals that can receive suspected cases are highlighted on the map so that people can find COVID-19 treatment hospitals. Map service providers also used mobile phone signaling data to measure the density of people in specific areas and differentiate the display on the map through red, yellow, and green color differences to remind the public of areas to avoid to reduce the risk of infection.

Based on the card swipe information data, systems developed by the public transportation management departments allowed passenger flow and full load of subways and buses data to be queried in a timely manner to select the best travel arrangements and times, to avoid congestion, and to reduce the risk of infection. The epidemic prevention department can quickly confirm the flight, train, or bus information of travelers infected with COVID-19 within the past 2 weeks and inform fellow travelers who have been in close proximity to be tested and take self-isolation measures. To inform the traveling public about high-risk travel, internet companies entered the information of flights, trains, or buses used by patients with COVID-19 into a tracking system and developed a “close contact measurement instrument.” By entering their ID number and name, a traveler can immediately check whether they have taken the same flight, train, or bus as a patient with COVID-19.

Through epidemiological surveys based on questionnaires, the staff of the epidemic prevention department can identify the areas and the specific times patients with COVID-19 visited before becoming sick and can promptly notify others who were active at the same time and place to pay attention to their health. For example, if a confirmed case occurs in a hospital, the hospital can notify other patients and people entering the hospital to isolate themselves through their mobile phone number. The operator’s mobile phone signaling data can timely and accurately identify the country where the user stayed in the previous 14 days and the domestic city where the user stayed for more than 4 hours to determine whether the user has been in a key epidemic area or country.

Most provinces and cities in China have established a big data health code system. When travelers scan their official Quick Response (QR) code, register, and fill in personal relevant information (including name; ID number; mobile phone number; body temperature; cities and travels in the past 14 days; and whether they have symptoms such as fever, coughing, and fatigue), the system will automatically generate a personal “health QR code,” which is divided into three color levels of red, yellow, and green [39]. Green means that the person did not appear in the virus-infected area during the quarantine period and can carry out cross-regional movement. When people with green codes have been to high-risk areas and in contact with high-risk people, their code will turn red. People with red or yellow codes will be quarantined, and the codes will turn green when certain conditions are met.

The digital epidemic prevention system, jointly developed by Alibaba’s Bodhidharma, Dingding, Alipay, and Alibaba Cloud, summarizes information from the hospital’s diagnosed cases and identifies people who have purchased fever-reducing drugs in pharmacies within the past month. Big data technology is then used to analyze the activity trajectories of confirmed cases and close contacts, and an epidemic spread model is constructed in combination with the positioning system. Based on the mobile phone positioning system of the diagnosed cases and close contacts, the expert team can find other mobile phone numbers within a 3-meter transmission range and more than a specified contact time based on the physical distance between the mobile phones to analyze the infection risks between parties. The big data positioning information can identify a large number of close contacts and quickly establish a province-wide big data outbreak early warning mechanism. For example, major epidemic transmission centers have been identified, including the Baodi Mall, a Tianjin hospital, the Tulong shopping mall in Harbin, and the Yintaidao shopping mall in Wenzhou [30].

### Medical and Health Data

For epidemic prevention and control, big data in health care can promote the timely detection and reporting of cases, improve the probability of finding diagnosis and treatment methods quickly, and improve the efficiency of hospital management in a pressure environment. There were initial failures in fully identifying and reporting COVID-19 cases in the EMRS disease reporting system. EMRS information should be complete and accurate. In the early stages of the epidemic, frontline doctors were not efficient in collecting patient data, identifying patients’ conditions, and reporting infectious diseases. One way of addressing this problem was to link EMRS data to epidemic decision making through China’s Center for Disease Control and Prevention (CDC), the government’s public welfare institution responsible for the technical management of disease control and public health [40]. By connecting the CDC’s monitoring and early warning system to hospitals’ EMRS, big data technology was applied in a timely manner to extract and analyze medical big data. First, through automatic matching, CDC’s AI knowledge base interrogated EMRS for keywords such as pneumonia. When suspected hospitals cases were identified, the EMRS immediately monitored front-end doctors’ computers, prompting doctors to verify the completeness and accuracy of the EMRS information and generate an infectious disease report. Such monitoring systems attenuate concealing or underreporting infections. Second, CDC monitoring improved the discovery and testing of EMRS big data to ensure the effective transmitting of health data and to avoid late or underreporting. For example, the CDC monitoring system reduced the average time required for doctors to report a COVID-19 case from the previous 5-8 minutes to within 40 seconds and the time required for online reporting via the CDC web-based infectious disease reporting system from 2-3 minutes to a few seconds [41]. CDC monitoring also supported COVID-19 diagnosis both within China and worldwide. One example was the successful isolation of the first COVID-19 virus strain in China, with the publication of the virus’ scientific information, electron micrographs, specific primers, and probes for global use. In the early stages of COVID-19 in China, the failure to provide timely data allowed the virus to spread both locally and worldwide. In the middle stages of the disease, timely release of scientific resources and data provided COVID-19 genes and gene sets, and big data information for COVID-19 research platforms for Chinese and international researchers [42]. CDC monitoring also supported hospital management. For example, the CDC promoted the integration of EMRS, HIS, and LIS data, and the hospital operations data to manage protective supplies, health status reports, epidemic developments, and telecommuting. By analytically and visually processing the integrated data, managers made decisions and assessments, which supported hospital treatment regimens [40]. When medical resources were overwhelmed by patients, big data technology helped develop a hierarchical diagnosis and treatment system, which allocated scarce health resources efficiently. In epidemic prevention and control, big data predictive analytics were mainly applied to predicting the impact of epidemic developments on medical resources. Based on big data models such as communication dynamics and risk level distribution of the incidence rate and close contacts, predictive analytics estimated epidemic peaks and inflection points, which allowed the differential allocation of resources to regional hospitals. Big data searches on internet platforms and susceptible-exposed-infectious-removed (SEIR) transmission modeling can predict COVID-19 transmission trends [43]. Using population migration data to fill in the dynamic propagation SEIR model combined with AI methods trained with SARS data, Yang et al [44] predicted a COVID-19 pandemic curve. The authors showed that if the Chinese authorities had postponed the implementation of strict public health measures for 5 days, the scale of the epidemic would have tripled. A loosening or removal of lockdown interventions would have caused Hubei Province to peak again from mid-March to late April [44].

Based on the predicted epidemic trends and risk level information, health departments deployed prevention resources in advance of the epidemic in specific areas to contain the spread of the virus and avoid second outbreaks. Relevant government departments also used big data predictive analytics to identify the epidemic’s peak and inflection point to determine the approximate time to resume normal work. Finally, big data predictive analytics was applied to integrate HIS medical costs and insurance information to estimate epidemic trends, proportion of patients of different histology types, the cost of diagnosis and treatment, and the allocation of health resources.

Further, big data diagnostic analytics can screen existing clinical pneumonia drugs to treat patients with COVID-19. For example, big data on patients prescribed traditional Chinese medicine were used to explore the efficacy of Chinese medicinal materials and their composition to treat COVID-19. Zhang et al [45] systematically screened natural compounds used in Chinese treatment and found 13 of them to exert potential anti–COVID-19 benefits in terms of the regulation of viral replication, modulation of immune and inflammatory pathways, and hypoxia cascade.

### News Media and Social Data

During the outbreak, there were inaccurate statements and misleading information on the internet, such as rumors that high alcohol content drinks and gargling with salt water could resist COVID-19, drinking Banlangen and smoked vinegar could prevent COVID-19, and that children could not be infected with COVID-19 [46]. Such false information will lead the public to relax their vigilance and take incorrect COVID-19 epidemic prevention measures. After identifying this type of false COVID-19 information, internet companies alerted epidemic prevention experts and national authoritative epidemic prevention organizations to take action to correct such false or inaccurate information. China took action to correct false information through authoritative national media, short films, and live broadcasts [47].

When a major epidemic occurs, the negative impact of uncertainty and panic on social activities may exceed the negative impact of viral diseases. Social media data can track and evaluate the spatial spread of public sentiment. By tracking topics and sentiments that appeared in microblog users’ timelines in response to COVID-19, a user semantic behavior evolution model was introduced to measure and analyze changes in public opinion [30,43]. The results indicated that from January 9 to February 10, 2020, more than 60% of posts related to science popularization of disease prevention and government COVID-19 announcements were positive and stable. For example, the posts with the topic of “help-seeking” were concentrated in the key epidemic area of Wuhan, and the posts related to “donation information” were widely distributed throughout the country [30].

Through real-time monitoring of keyword information searched by users, such as the frequency of the words “fever,” “epidemic,” “cough,” “pneumonia,” and “infection” on internet platforms, health officials assessed the health status of people in various regions. When the frequency of such keyword searches increased rapidly in a certain area, the risk of a large-scale outbreak in that area was increased accordingly [5]. Such tracking allowed relevant health departments to formulate epidemic prevention and treatment measures in advance. For example, Qin et al [43] used big data to predict the number of suspicious or confirmed new cases of COVID-19. Using a series of lagging “social media search indexes” for various keywords including clinical symptoms of COVID-19 (such as dry cough, fever, chest pain, and pneumonia), the authors found that COVID-19 outbreaks could be found 6-9 days in advance.

### Data Collected by Intelligent Equipment

Using thermal imaging human body temperature measurement equipment in public places such as train stations, airports, shopping malls, hospitals, and pharmacies to automatically collect data, individuals with an abnormal body temperature in a large number of passing people can be identified using face recognition technology [48]. Once identified, people with COVID-19 can be isolated, containing the spread of the virus. This big data early warning system can automatically collect and analyze information to diagnose people with COVID-19 in real time.

### Data on Epidemic Prevention Materials

The spatial distribution of medical resources is usually balanced according to factors such as population density, but the uneven spatial outbreak and spread of COVID-19 creates large regional imbalances in the supply and demand for medical resources. The key to epidemic prevention and control is to understand the spatial and temporal dynamics of the supply and demand of medical resources to optimize the distribution of materials, resources, and medicine. Sharing data with emergency suppliers and public resource national trading institutions underpins a system that provides information on supply and demand for resources and maintains unified prices to minimize unfair competition, ensure material security, and guarantee material quality [49]. Through telephone and online inquiries, the dynamic status of medical protection equipment in China was analyzed. Data analytics on material needs identified the demand from multiple locations and allowed health organizations to coordinate the release and tracking of medical supplies by their name and quantity of required materials, contact information, and transport mode [50]. By stopping the release of material supplies through scattered, uncoordinated channels, big data allowed the managed allocation of supplies to hospitals and cities with the greatest need, balanced supply and demand, and ensured the efficient allocation of medical resources.

A stable and efficient national material supply and transportation system provides important support for successful epidemic prevention work. For example, using provincial epidemiological data, online consumption data and postal service data identifies each provinces’ supply and demand status for necessities and food, preventing shortages and price changes [51]. At the same time, by tracking the transportation of materials, highly sensitive nodes that may cause virus transmission during transportation were identified, and early warning and decision support were provided to prevent and control the regional spread of COVID-19.

### Online Consumer Data

Big data can help enterprises to target production, marketing, logistics, and safe work resumption. Amid the epidemic, enterprises producing medical products and basic necessities can assess public demand using big data to ensure supply and demand equilibrium by scheduling optimal production and efficient distribution of products. Big data, for example, identified electric pressure cookers as one of the most talked about products during the epidemic, which allowed firms to target the production and distribution of pressure cookers [52].

### Government Data

During the epidemic, the use of big data technology for population information management and decision making was a fundamental approach by government service departments [53]. Tax data, especially value-added tax invoice data, have the unique advantages of wide coverage, strong timeliness, and fine granularity, helping to schedule the resumption of work, production, and the operation of enterprises [54]. Gathering data on industry, region, scale, and economic type, national and local governments analyzed the dynamics of the economy’s operation, planned the resumption of work, determined the production levels and sales of enterprises, identified and solved problems along the industrial supply chain, and provided targeted assistance to individual business and business sectors. For example, the Shandong Provincial Taxation Administration used big data to help a local pharmaceutical glass company to distribute their epidemic prevention supplies by screening logistics companies that had a high degree of credibility and carrier capacity, ensuring the smooth delivery of their supplies [55].

Through big data analysis, governments can assess the impact of the epidemic on socioeconomic operations in the near and long term; establish economic emergency response systems; implement various incentive measures such as tax cuts, fee reductions, and special subsidies; mitigate the risk of financial chain breakage; and promote the continuous operation of small- and medium-sized enterprises. For example, the Shanghai Municipal Tax Administration used tax data to identify potential targets for tax incentives and subsequently launched more than 30 tax incentive programs, covering more than 200,000 tax companies [56].

### Deep Learning Data

Used in a variety of fields, deep learning is a machine learning analytic method frequently used to interrogate big data, performing tasks that are difficult for conventional analysis methods [57-60]. Mobilizing big data, deep learning offers a method to better forecast disease trends, including COVID-19. For Hong Kong, Xu et al [61] showed deep learning yielded better prediction performance for flu-like diseases than the generalized linear model, the least absolute shrinkage and selection operator model, and the autoregressive integrated moving average (ARIMA) model. Wang et al [62] found deep learning provided a convenient tool for fast screening COVID-19 and finding potential high-risk patients, which may be helpful for medical resource optimization and early prevention before patients show severe COVID-19 symptoms. Various non-China studies indicate that infectious disease can be predicted more effectively when large amounts of data including weather variables and internet big data are used to predict infectious diseases. Using deep learning, Chae et al [63] found that deep neural network and long-short term memory learning models outperformed ARIMA approaches when predicting for three infectious diseases in Korea 1 week into the future. Using data mining algorithms to predict COVID-19 outbreak trends, Ayyoubzadeh et al [8] showed that the most effective predictive factors, besides the previous day incidence, was the search frequency of handwashing, hand sanitizer, and antiseptic topics. Togacar et al [64] found a deep learning model using data on COVID-19, pneumonia, and normal x-ray imaging could detect COVID-19 efficiently.

## Discussion

### Issues and Challenges in COVID-19 Big Data

#### Data Collection and Access

During a pandemic, the way data are collected and the quality of the collected data pose challenges. When data were collected by government workers exposed to disease environments, causing physical and mental dangers, data standards were not uniform. For organizations, different information reporting systems and data reporting formats were adopted, resulting in inconsistent standards, unreliable structures, and untimely reporting, especially in the process of statistical aggregation [65]. Frequently, data were not updated in a timely manner. Against the backdrop of the rapid spread of the COVID-19 virus, immediate data updates were essential to assess the state of the epidemic and its dangers, and to avoid panic among the public. However, due to insufficient awareness of the epidemic’s risk in the early stages, the lack of advanced means of data collection, the large number of subjects involved in data collection and release, and the inevitable “fragmentation” of big data platforms, much of the data were not updated timely or optimally.

#### Data Use

Data use depends heavily on visualization, and data mining techniques were found inadequate. Data source, quality, and scale all influence data integration, and raw data collection issues contribute to the difficulty of data integration. Converting low-value density big data into high-value density knowledge through data integration is the purpose of big data management applications [66]. Given existing data development capabilities, there has been a general lack of effective integration of data sources. This has been complicated by the rapid development of internet and information technology, and the rapidly expanding types and volume of data resources. Data quality and degree of data analysis determines big data’s role in epidemic prevention and control. Much of the data used for epidemic prevention and control were available through official channels or accumulated by enterprises during their operations but were of widely varying quantity, quality, and variety. It is often the case that data holders do not have sufficient skills to exploit good data, or those with good data development skills do not have sufficient data.

The visual presentation of spatial, temporal, and quantitative features of epidemic data in the form of maps is the most common means of using epidemic big data. Epidemic surveillance is about spatial governance through spatial-temporal modeling and analysis, including the control of people flow, logistics, information flow, and technology flow. Although geographic information technology has unique advantages in data collection, processing, and management, most visualization products simply present, rather than analyze, information about the epidemic. In the immediate COVID-19 period, there were insufficient data on transportation networks, service facilities, public opinion, prevention and control effects, and the information on their mutual relationship. More importantly, there was inadequate innovation in the way the data were used.

#### Data Sharing

The key to the role of digital government and smart cities in managing major public emergencies is to enable data sharing. In 2015, China’s State Council issued the *Action to Promote the Development of Big Data* [67], which proposed that big data should be used as an important means to enhance the government’s governance capacity, improve the level of government decision making, and manage risk prevention through efficient collection and integration of government and social data. By the end of 2017, China had introduced a series of policies to promote effective government data sharing and completed the construction of a national data sharing platform to promote the sharing of data resources at the provincial and ministerial levels. In practice, data sharing has displayed a silo effect. Data sharing among Chinese departments and regional governments remains inadequate, and the phenomenon of “block islands” still exists. For example, the health code is a two-dimensional risk assessment code generated from personal travel information and health status information. During the use of these health codes, some regions did not recognize the health codes generated by other regions [39]. Partly, this is due to inadequate infrastructure for urban data collection, lack of community data resources, and lack of big data management expertise, which led to a disconnect between spatial and social governance. However, there was a continuing unwillingness to share data across provinces and municipalities, and insufficient means of information interaction, resulting in inaccurate data analysis and weak regional joint prevention capacity. Only by fully realizing data flow across regions, levels, and sectors can data be efficiently integrated and better applied to epidemic management.

#### Data Protection

In terms of data protection, there is inadequate privacy protection and undisclosed compliance risks. The value of big data lies in its disclosure and concrete application. In the age of the internet and electronic information, people’s lifestyles generate big data on all aspects of their life, but privacy may be compromised. In the face of the rapidly spreading epidemic, government investigates and closely monitors the health of individuals, collecting a large amount of personal information on COVID-19 confirmed, suspected, and potential cases and their close contacts. Information on people leaving Wuhan to visit their home villages and cities have been exposed on the internet, which caused a serious negative impact on individuals and families who were subjected to unwarranted harassment, discrimination, and physical threats [68].

### Strategies and Recommendations

#### Data Collection and Access

From China’s big data experience during the first stages of the COVID-19 pandemic, data collection should have been more timely, comprehensive, and accurate. To improve data collection and access, electronic questionnaires should be promoted to conduct information surveys, including the use of the WeChat applet or Alipay applet. For COVID-19 tracking, after scanning their QR code, individuals can efficiently input their information such as ID card number, household registration, residential address, and itinerary of the past 2 weeks, and then update their body temperature every day. Of course, manual collection of data can lead to delays, concealment, inaccuracies, and underreporting. One solution is to collect these data automatically by apps, which will improve data accuracy and use. Accurate tracking data can be collected using portable electronic equipment, cameras, access control, license plates, and other information.

To conduct more in-depth scientific analyses, data on weather, news, transportation, business, and health are currently collected in addition to collecting accurate personal information data. The credibility of these data is important. For example, when the mobile phone number registered according to an individual’s ID card information is used by others, there will be errors in predicting a person’s movements. In addition, individuals deliberately concealing their own information or falsely reporting their own information leads to data errors. Data sharing will allow cross-comparisons of different data sources, including mutual authentication of government, operator, traffic, public security, network, and other information. For example, the National Health Commission and the national information security department could work together to formulate common standards of data structure, format, attributes, content, and scope for individuals, and release these data to the institutions or departments with investigation rights to ensure the quality of the data. Cross-comparisons of data raise issues of privacy and whether intentionally concealing or falsely reporting data should be legally punished.

#### Data Use and Sharing

Data use and sharing require the integration of data sets and the strengthening of data sharing. One way to integrate data is to give full play to the technical advantages of scientific research institutions or commercial units. For example, the functions of the Guiyang big data exchange center, Shanghai Data Trading Center, and commercial data package trading platforms (such as COFCO, Youyi, Yaxin, and shuduoduo) can integrate, use, and share data. In addition, one way to innovate data use is to use blockchain technology to build a neutral third-party security housing platform to realize the separation of ownership and use rights [69]. Secure house platforms allow the demand side to use data that it does not own. For the data supplier, secure house platforms provide massive data access to multiple data sources. For the data demander, it uses data fusion technology to provide multiparty high-quality data resources that the data demander did not have previously. In addition, it supports the third-party algorithm and user-defined algorithms. For example, AI companies can carry out AI analysis and algorithm modeling for pathological sections of patients in the secure house, which not only ensures patients’ privacy and prevents the leakage of medical data but also optimizes the use of the AI. To fully realize big data sharing in a secure way, governments can set an identity for big data assets and establish a data query mechanism to realize cross-platform data tracing and extraction.

#### Data Innovation

The value and efficiency of big data use can be improved by making full use of big data mining and data analysis methods. Based on big data resources and big data analysis methods such as machine learning and data simulation, a more comprehensive and rapid assessment of the risk and impact of the epidemic could be carried out. Strengthening data collaboration, for example, between data owners and big data technology platforms, and integrating multidisciplinary knowledge and analysis methods will yield innovative big data solutions. The health code is a successful example. Information on individuals’ ID, residential address, health status, whether they have been to the infected area, and contact history of key personnel were entered into the applet and a QR code generated. Spatially, the risk level of the epidemic in defined areas were assessed temporally by the number of visits, length of stay, and interpersonal relations between individuals in epidemic areas, allowing three risk states (red, green, and yellow) to be measured, analyzed, and assessed [39].

A further innovation would be the expanded use of the QR code, where individuals would be required to scan their QR code to show their health code status before entering public places. At the same time, the health code would also record the current position of the individual. When their QR code is red or yellow, they would be denied entry to public areas. In addition, when there are COVID-19 cases in specific public areas, the health department can quickly inform individuals not to enter or to isolate through information automatically reported by their health code. In addition to obtaining basic health information of residents during the epidemic, the health code can obtain medical insurance information and other disease information, realize a series of digital services in the hospital system from registering appointments to picking up medicines, and fully exploit new opportunities for online appointments and online consultations while accumulating data assets for the analysis of medical and health big data intelligence applications.

#### Data Security and Privacy

Attention should be paid to big data privacy issues [70]. Privacy issues are fundamentally country-specific legal requirements, where China’s privacy framework will vary from that of other countries. Guarantees of nondisclosure and proper use of personal privacy information are the most basic requirements for data collection and use. Statistical processes involving the collection, aggregation, sharing, and disclosure of personal information should require the protection of personal information to prevent data leakage, loss, and abuse. Governments should establish institutional safeguards, listing situations that require the public to declare private information data. The government should also specify the list of institutions that can request personal privacy information and the requirements allowing third-party access to personal data. In China, the law on the prevention and control of infectious diseases stipulates that “any unit or individual within the territory of China must accept the prevention and control measures such as the investigation, inspection, collection of samples, isolation and treatment of infectious diseases by the Department of Disease Prevention and Control, and truthfully report the relevant information” [71]. On February 9, 2020, China issued a notice protecting personal information and regulating the use of big data to support joint prevention and control of diseases, specifying that personal information collected for epidemic and disease prevention and control should not be used for other purposes. No unit or individual may disclose information such as name, age, ID card number, telephone number, or home address without the consent of the collector, except for those who have been desensitized due to the need for epidemic prevention and control [72]. Government departments only release information of public concern, such as “digitizing” individual patients. Each confirmed patient has a code, which only shows their gender, age, disease severity, and frequent activity area. There are legal penalties for failing to follow the legal requirements on data handling.

Privacy concerns might be further addressed by enhancing big data’s privacy framework, including setting access and use permissions for the encrypted data collected, making the data available only to institutions or organizations with a clear purpose, ensuring only the minimum personal information for epidemic prevention and disease control is collected, and setting retention periods that limit the time collected personal information is retained.

Big data helped contain, suppress, and control COVID-19. The main lesson is that COVID-19 big data technology can be applied to epidemic alerts, tracking the spread of viruses in real time, planning public health interventions, monitoring the effectiveness of treatment regimens, identifying potential vaccine candidates, strengthening community and regional government responses to epidemics, and guiding social and economic recovery in the postepidemic period. In the long run, big data technology helps build smart, healthy, and resilient cities and countries.

### Conclusion

In China, big data technology displayed its potential in containing, suppressing, and controlling COVID-19, with the Chinese government and nongovernmental organizations making full use of big data information from the major areas of medical institutions, telecommunication operators, internet companies, government departments, commercial companies, and AI equipment. Through the analysis of big data technology to prevent and control COVID-19, we found that big data technology played an important role in epidemic surveillance and early warning, tracing of virus sources and personal tracking, planning public health interventions, monitoring drug screening and medical treatment, strengthening community and regional government responses to epidemics, and guiding social and economic recovery in the postepidemic period. These big data applications were central for the public, enterprises, and governments in their response to COVID-19.

We also identified gaps in the use of big data technology to prevent and control COVID-19, including problems in data acquisition, data use, data circulation, and data protection. The application of big data technology in the prevention and control of infectious diseases like COVID-19 requires further development in the collection, analysis, distribution, and privacy around personal and public information. Although China’s big data application to fighting the COVID-19 epidemic was a success story, it also provides lessons for the continuous improvement in the application of big data technology to epidemics in China and for other countries managing the COVID-19 pandemic.

## Abbreviations

AI: artificial intelligence
ARIMA: autoregressive integrated moving average
CDC: Center for Disease Control and Prevention
EMRS: electronic medical record system
HIS: hospital information system
LIS: laboratory information system
NIDRIMS: National Infectious Disease Report Information Management System
QR: Quick Response
SARS: severe acute respiratory syndrome
SEIR: susceptible-exposed-infectious-removed
